# Physicochemical Compatibility of Ceftolozane-Tazobactam with Parenteral Nutrition

**DOI:** 10.3390/ph17070896

**Published:** 2024-07-05

**Authors:** Jan Thomas De Pourcq, Adria Riera, Laura Gras, Noe Garin, Maria Antònia Busquets, Joana Cardenete, Daniel Cardona, Pau Riera

**Affiliations:** 1 Department of Pharmacy, Hospital de la Santa Creu i Sant Pau, Universitat Autònoma de Barcelona, 08025 Barcelona, Spain; 2 Department of Medicine, Universitat Autònoma de Barcelona, 08025 Barcelona, Spain; 3 Institut de Recerca Sant Pau (IR SANT PAU), 08041 Barcelona, Spain; 4 Department of Pharmacy, Hospital de Sant Pau i Santa Tecla, 43003 Tarragona, Spain; 5 School of Health Science Blanquerna, Universitat Ramon Llull, 08025 Barcelona, Spain; 6 Centro de Investigación Biomédica en Red de Salud Mental (CIBERSAM), Instituto de Salud Carlos III, 28007 Madrid, Spain; 7 Institute of Nanoscience and Nanotechnology (IN2UB), University of Barcelona, 08028 Barcelona, Spain; mabusquetsvinas@ub.edu; 8 Department of Pharmacy and Pharmaceutical Technology and Physical-Chemistry, Faculty of Pharmacy and Food Sciences, University of Barcelona, 08028 Barcelona, Spain; 9 CIBER de Enfermedades Raras (CIBERER), Instituto de Salud Carlos III, 28029 Madrid, Spain

**Keywords:** ceftolozane-tazobactam, critical care, parenteral nutrition, PN, compatibility

## Abstract

Ceftolozane-tazobactam (CT) is used for the treatment of complicated infections and for multidrug-resistant strains of Pseudomonas aeruginosa and extended-spectrum beta-lactamase-producing enterobacteria. In certain cases, simultaneous administration of CT and parenteral nutrition (PN) may be required, but compatibility of Y-site co-administration is unknown. The aim of this study was to analyse the physicochemical compatibility of CT Y-site administered with PN. We evaluated a protocolized PN approach for critical patients in our center. We studied both bolus infusion (2 g ceftolozane/1 g tazobactam in 1 h) and continuous infusion (CI) (6 g ceftolozane/3 g tazobactam) strategies. Samples were visually observed against light, microscopically inspected, and pH was analysed using a pH meter. The mean lipid droplet diameter (MDD) was determined via dynamic light scattering. CT concentration was quantified using HPLC–HRMS. No alterations were observed through visual or microscopic inspection. Changes in pH were ≤0.2, and changes in osmolarity were less than 5%. MDD remained below 500 nm (284.5 ± 2.1 for bolus CT and 286.8 ± 7.5 for CI CT). CT concentrations at t = 0 h and t = 24 h remained within prespecified parameters in both infusion strategies. CT is physiochemically compatible with PN during simulated Y-site administration at the tested concentration and infusion rates.

## 1. Introduction

Ceftolozane-tazobactam (CT) is the combination of a novel cephalosporin and a beta-lactamase inhibitor. Approved by the FDA in 2014 and by the EMA in 2015, it was initially indicated for the treatment of complicated intra-abdominal infections, acute pyelonephritis, and urinary tract infections in adults. Later approvals extended its use to hospital-acquired pneumonia, including pneumonia associated with mechanical ventilation [1]. This novel antibiotic has become an option for treating multidrug-resistant Pseudomonas aeruginosa strains and extended-spectrum beta-lactamase-producing enterobacteria. Given the escalating global health threat posed by antibiotic resistance, its use is expected to rise in the coming years. In effect, off-label indications are frequently used in clinical practice for skin and soft tissue infections, osteoarticular and intra-abdominal infections, and bacteremia [2]. The recommended dosing regimen is 1 or 2 g of ceftolozane administered via intravenous infusion for one hour every 8 h [1,3,4]. Patients who receive CT usually have infections caused by multi-resistant microorganisms. Due to the limited alternatives available and to avoid resistance, it is essential to ensure therapeutic effectiveness. As with other beta-lactams, the objective in relation to efficacy is to sustain plasmatic concentrations above the minimum inhibitory concentration (MIC) throughout the dosing interval.

In critically ill patients, treatment of bacterial infections can be particularly difficult due to the possibility of multi-organ failure and hemodynamic, physiological, and metabolic alterations. Numerous factors can affect pharmacokinetics, such as hyperdynamic states, intensive pharmacotherapy, fluid overload, increased or impaired renal clearance, and even support with continuous renal replacement therapy or extracorporeal membrane oxygenation. Standard dosage regimens may fail to achieve therapeutic levels. For all these reasons, especially in critically ill patients or when dealing with high MICs, it is recommended to administer the daily dose of CT via extended infusion (over 3–4 h) or continuous infusion (over 24 h) [5,6,7,8].

In intensive care units, decreasing the risk of medical errors is imperative, particularly those related to the administration of incompatible drugs through the same infusion line. Patients in these units frequently require complex therapeutic regimens, often including multiple continuous infusions and parenteral nutrition (PN). Given the limited availability of venous access, the Y-drug delivery strategy is often used to allow multiple concomitant administrations. However, this approach should be avoided when possible as it poses inherent risks. An alternative to co-administration is the temporary interruption of the supply of the PN admixture. However, vascular manipulation may increase the risk of infection and provoke glycaemic alterations [9].

In the critical care setting, clinical nutrition should be considered within the first 48 h. PN is indicated when enteral or oral nutrition is not feasible or does not fulfill patient requirements [10,11,12]. Ensuring the appropriate use of PN is crucial to maximize its benefits while minimizing the risk of complications. PN is a highly complex drug. It contains more than 50 possible ingredients and carries a risk of interaction. It is a high-alert medication that requires safety-focused policies, procedures, and practices to mitigate potential hazards and optimize therapeutic outcomes. Additionally, PN compatibility and stability are affected by external factors such as pH, temperature, oxygen, light, and infusion sets or containers, which should be taken into account [13,14].

Drug Y-site compatibility fundamentally depends on a drug’s physicochemical characteristics, but the concentration, temperature, and light exposure may also play an important role. In some cases, instability between mixtures can be visualized through color changes, phase separation, or the formation of precipitates. Precipitates of particles larger than 5 μm can lead to complications such as reduced drug bioavailability and occlusion of blood vessels. Such complications can be fatal, either due to a lack of therapeutic effect or by triggering the formation of a thrombus [15,16,17]. It is, therefore, essential to ensure compatibility between solutions that will come into contact with PN at site Y. Many studies and reviews have focused on the compatibility of antibiotics with PN [18,19,20,21,22,23,24,25], but compatibility has not been elucidated for many other drugs. The aim of this study was to evaluate the physicochemical stability of CT when mixed with a protocolized PN emulsion during Y-site administration.

## 2. Results

### 2.1. Physical Stability

Visual inspection of PN emulsions did not show any macroscopic precipitates. There were no signs of phase separation, color changes, or gas formation at t = 0 h and t = 6 h.

Microscopic inspection showed no evidence of incompatibility. No precipitates or presence of particles >5 µm were detected.

Similarly, for PN-CT admixtures, no alterations were observed via visual inspection in any of the CT concentration ranges at t = 0 h and t = 6 h.

The average pH of bolus CT was 5.77 ± 0.05, and the average pH of continuous infusion CT was 5.68 ± 0.02. The pH of PN was 6.28 ± 0.02 at t = 0 and 6.14 ± 0.01 at t = 24 h. The average pH of the admixtures in bolus conditions was 6.18 ± 0.03 at t = 0 and 6.06 ± 0.02 at t = 24. In continuous infusion conditions, the pH was 6.23 ± 0.01 at t = 0 and 6.10 ± 0.01 at t = 24 h.

MDD was around 290 nm, which is lower than the 500 nm limit accepted by the US Pharmacopeia in all samples. The mean MDD change was less than 5 nm and less than the prespecified Δ10%: for PN, it was 282.3 ± 5.6 nm; for PN with bolus CT, it was 284.5 ± 2.1; for CI CT, it was 286.8 ± 7.5 (Table 1) (Supporting Materials Figures S1–S3).

The mean osmolality of the PN solutions was 1935 ± 20 mOsm/kg at t = 0 h and 1921.6 ± 18.8 at t = 6 h. In bolus conditions, osmolality was 1103.6 ± 11.5 mOsm/kg at t = 0 h and 1087.6 ± 5.3 at t = 6 h. In CI conditions, the osmolality was 1748.1 ± 15.6 mOsm/kg at t = 0 h and 1722.4 ± 9.5 mOsm/kg at t = 6 h. All osmolality determinations fulfilled the criteria of less than a 5% change. Table 2 shows the average osmolality changes at t = 0 h and t = 6 h.

### 2.2. Chemical Stability

The average concentration of ceftolozane in bolus administration at t = 0 h was 8.43 ± 0.34 mg/mL, and at t = 24 h, it was 9.37 ± 0.51 mg/mL. In CI administration, the average concentration at t = 0 h was 1.57 ± 0.19 mg/mL, and at t = 24 h, it was 1.50 ± 0.26 mg/mL. In the case of tazobactam, the average concentration in bolus administration at t = 0 h was 4.3 ± 0.13 mg/mL. At t = 24 h, it was 4.21 ± 0.24 mg/mL, and in CI administration, at t = 0 h, it was 0.89 ± 0.16 mg/mL. At t = 24 h, it was 0.87 ± 0.15 mg/mL. In bolus conditions, the mean changes in ceftolozane and tazobactam were +11% and −2%, respectively, and in CI conditions, the mean changes were −5% and −2%. Table 3, Table 4 and Table 5 show the concentrations of CT and the ceftolozane–tazobactam ratio at t = 0 and t = 24 h.

## 3. Discussion

Our results show that CT co-infusions, either as bolus or CI, are stable over time when they are infused in a Y-site alongside PN. To the best of our knowledge, Y-site compatibility studies have not been conducted to assess the concurrent administration of CT and PN.

When multiple IV infusions must be administered simultaneously, they should be infused separately, if possible. However, some patients require complex prescription regimens that can be a challenge when Y-site administration is inevitable. Drug–drug incompatibilities should be assessed to avoid safety issues that may result in precipitation or occlusion of infusion lines [26], which could ultimately lead to serious consequences, such as venous thrombosis or pulmonary embolism. However, there is limited research available regarding the stability of PN when co-administered with other drugs.

Many factors, such as PN composition, storage conditions, and administration practices (e.g., bag type, temperature, administration set, and use of filters), can affect PN stability results. To avoid these possibilities, we simulated administration conditions following standard protocols and the recommendations for PN and CT administration. In the literature, two methods are described to assess compatibility [27]: static methods combining medication in tested ratios in a test tube and dynamic methods simulating their co-administration with automatic pumps. In the present study, we simulated the second method as it reflects clinical conditions and takes the influence of relevant factors such as infusion line characteristics into account.

In our study, we used a standard PN composition protocolized in our center. This protocol can be used both in stable and critical patients, according to guidelines [10,11,28,29]. The PN formulation included standard electrolyte concentrations as well as trace elements and vitamins in order to resemble usual conditions that could affect stability and accelerate the decomposition of the drug. We tested PN with fish oil (FO) containing intravenous lipid emulsions because FO emulsions have been associated with more clinical benefits, particularly in the critical care population [30,31]. Our PN could also be considered for use as supplemental PN in critical patients who have high energy and protein requirements. However, when assessing PN composition, it should be kept in mind that critical care patients may present conditions or complications that warrant the use of individualized PN compositions other than the tested PN.

When considering drug characteristics, various factors, such as product excipients, carrier fluids, final drug concentration, and contact time of exposure, should be taken into account. According to the SPC [1], reconstituted CT can be diluted in normal saline (NS) or 5% glucose solutions. In our study, we diluted CT in NS. To our knowledge, this is the most common infusion fluid used in the preparation of drugs in the intensive care ward. The use of 5% glucose solutions is generally avoided so as to prevent an excessive supply of glucose, which could lead to glycemic changes. Moreover, in a study analyzing Y-site administration of CT and selected intravenous drugs, the authors found that compatibilities and incompatibilities were consistent regardless of whether NS or 5% glucose was used as a diluent [32].

The tested antibiotic concentrations in the present study may help assess the possibility of co-administration under different therapeutic strategies, both of which can result in different proportions of medication coexisting within the same infusion line. Although it is not described in the SPC, the CI strategy is commonly used in critical patients because it provides more stable concentrations of the drug. This approach has been described previously [1,33,34]. However, such use outside the terms of its marketing authorization is not supported by the manufacturer.

There remains a lack of consensus regarding the specific tests that should be performed to assess compatibility between drugs and PN. The methods we selected were based on our previous experience [17,35,36], a literature review [15,19,20,22,24,25,37], and the instrumental techniques available in our area that could be performed without excessive temporal delays.

The assessment of physical compatibility was based on visual inspection and measurement of pH, osmolality, and lipid emulsion particle size. These parameters were assessed immediately after preparation and again between 6 h and 24 h, depending on the availability of instrumental techniques. It must be taken into account that the contact time between PN and the tested drug is a few minutes and that the time between the second measurement could have been even shorter than the 6–24 h schedule. On the basis of the ILE by European Pharmacopeia criteria, we consider our methods to detect any signs of precipitation or emulsion destabilization [38].

One study reported incompatibility between CT and propofol, showing a phase separation in the oil emulsion with a free layer of oil [32]. This study also reported incompatibility with other drugs assessed with turbidity changes, a method that is not feasible in lipid emulsion or PN. However, stability changes should not rely only on visual inspection to define the safety of drug co-administration. Small particles cannot be detected because of the viscosity, rheology, or opalescence of PN admixtures and the proportion and size of the possible particles that should be detected.

The lack of pH and osmolality changes observed outside the prespecified ranges suggests the compatibility of CT with PN. Variations in these parameters that exceed the limits could indicate acid–base changes due to lipid hydrolysis with the release of free fatty acids or precipitation. It has been reported that at pH < 5.5, lipid emulsions can undergo phase separation, depending on the composition and concentration of electrolytes [16,19,24].

Lipid droplet size is one of the most relevant parameters when considering PN admixture safety [39,40]. The European Pharmacopeia does not propose size determination or limits related to the particle size of lipid emulsion [38]. Conversely, the US Pharmacopeia recommends two possible methods to assess this aspect: (a) light scattering and (b) light obscuration or extinction [41]. In our study, we applied the dynamic light scattering (DLS) method, with the MDD limit defined as 500 nm and with changes in MDD ≤ 5 nm, as reported in previous works [17,35,36] and applied by other authors [19,21,22,23,24,42]. Both in bolus and continuous infusion, our sample met the criteria and was within the US Pharmacopeia requirements.

Some authors consider the PFAT-5 parameter to be more reliable than MDD as it allows for a complete characterization of lipid droplets [43]. Several studies have assessed compatibility using light obscuration methods, measuring the percentage of large diameter droplets (>5 µm) that should not exceed 0.05% [27]. However, many other studies have assessed compatibility without testing this parameter [18,20,21,22,23,24,25,42,44,45]. Unfortunately, light obscuration was not available in our region. Nonetheless, we found a low SD and a low polydispersity index, indicative of homogeneous preparations without precipitates. (Supporting Information Figures S1–S3). Moreover, microscopic inspection was performed to rule out the presence of particles invisible to the naked eye.

Consensus is also lacking regarding the determination of drug concentration changes in Y-site administration. In our study, we simulated the co-administration of the drugs and considered a contact time greater than the real-time between the drug and PN in real clinical practice, which is only minutes. In bolus conditions, the mean changes of ceftolozane and tazobactam were +11% and 2%, respectively, and in CI conditions, they were +5% and +2%. In some samples, we observed changes in ceftolozane concentrations greater than 10% at 24 h. In the case of tazobactam concentrations, changes were less than 10%. The relationship between the ceftolozane and tazobactam concentrations in our study remained at a 2:1 ratio. Many studies have not assessed these changes because of the short contact time in the case of Y-site administration [19]. Findings from studies that have evaluated the drug in a PN admixture have suggested that the formulation should retain at least 90% of the active substance [18,22,24]. Aeberhard et al. [15] considered a larger deviation range of 20% acceptable because of the high complexity of the samples.

In a recent review of compatibility studies of PN and drugs for pediatric patients, Gostyńska et al. [27] proposed a grading system to assess the physicochemical compatibility data inspired by the Stabilis 4.0 database. They categorized studies into four groups (A–D) based on the quality and evidence provided. According to their system, our study would be categorized as grade C, indicating medium evidence, as we did not test PFAT through light obscuration or light extinction methods. However, we did assess chemical stability, a criterion for the A grade.

The main limitation of our study is that the results cannot be extrapolated to the dilution of the drug in the bag. However, we simulated the most common situation in clinical practice, which is Y-site administration. A second limitation is the need for a 1:10,000 dilution when processing samples for HPLC–HRMS determination. This dilution could account for the difference in concentrations of antibiotics in some of our samples, which showed variability above 10%. A third limitation could be the many possible PN compositions. To avoid this, we designed a PN according to ICU patient requirements. We used the most feasible formulation available in our center, based on standard amino acids, an omega-3 enriched lipid source, and requirements that fulfill the guidelines. Differences in PN composition when formulating individualized PN, with specific changes adapted to patients’ needs, should be taken into account when assessing compatibility administration recommendations. In such cases, our compatibility results cannot be inferred for all possible formulations. Another limitation was that droplet size was only analysed using MDD. Evaluating droplet size with light obscuration technique would reinforce the validity of the results. Finally, PN administration should be performed with the use of filters in order to increase safety by retaining possible particles >2 µm, which appear to present a higher risk for adverse consequences [46].

## 4. Materials and Methods

### 4.1. General Procedures

Three protocolized PN emulsions with the same composition and six CT solutions (three for bolus infusion and three for continuous infusion) were prepared in the Pharmacy Department at our centre. The emulsions were compounded in horizontal laminar flow hoods using ethyl vinyl-acetate (EVA) multi-layered bags (Oiarso S. Coop, Hernani, Spain). CT solutions were prepared under non-aseptic conditions, simulating regular clinical practice in the ICU setting. Bolus infusion and continuous infusion were simulated in Y-site administration with PN, as previously described [17,32]. Tests were performed in triplicate for each experiment.

### 4.2. Composition of PN Emulsions and CT Solutions

The three PN tested were equally compounded. The PN was designed to fulfill the requirements of a critically ill adult patient weighing 60–70 kg for a 24-hour period [10,11,29]. The composition of the PN was 14 g of nitrogen, 250 g of dextrose, 50 g of lipids, 80 mmol of sodium, 60 mmol of potassium, 60 mmol of chloride, 4.6 mmol of calcium, 5 mmol of magnesium, 50 mmol of acetate, 23.8 mmol of phosphate, trace elements, and vitamins (PN volume: 1615 mL). Specifically, we selected SMOFlipid^®^ 20% (Fresenius Kabi, Barcelona, Spain) as the lipid source and Aminoplasmal^®^ (B.Braun, Barcelona, Spain) as the nitrogen source (Table 6).

For the preparation of each of the three CT bolus infusion solutions, we reconstituted two CT 1000/500 mg (Zerbaxa^®^, MSD, Haarlem, Holland; Series expiration dates: W019837-03/2025 and W033928-03/2025) with 10 mL of water for injection and diluted in 100 mL of normal saline (NS) (Fisiológico B. Braun 0.9%, B.Braun, Rubí, Spain). The predicted ceftolozane concentration of these solutions was 16.3 mg/mL. For the preparation of each of the three CT continuous infusion solutions, we reconstituted six CT 1000/500 mg diluted in 250 mL of NS. The predicted ceftolozane concentration of these solutions was 18.8 mg/mL. Reproducing daily clinical practice, we did not remove the equivalent volume of the diluted drug from NS.

### 4.3. Simulation of Y-Site Administration

PN emulsions and CT solutions were infused as in clinical practice by means of automatic pumps (Infusomat^®^ Space, B.Braun, Rubí, Spain). The PN rate was 67 mL/h, simulating 24-h infusion.

To select the CT infusion rates to be tested, we followed the Zerbaxa^®^ SPC [1] and the bibliographic review [2,3,33,34]. For bolus simulation, the diluted drug was administered at a rate of 123 mL/h, and for continuous infusion simulation, the rate was 13.3 mL/h.

To simulate administration at the Y-site, we used opaque infusion sets. Both PN and CT preparations were protected from light.

### 4.4. Sample Collection, Storage, and Analysis

We simulated Y-site administration for 30 min both for bolus and continuous infusions and collected the final volume resulting from the admixture of CT and PN. The concentrations predicted in the admixtures were 9.75 mg/mL of ceftolozane and 4.85 mg/mL for tazobactam in the bolus simulation, and 2.4 mg/mL of ceftolozane and 1.2 mg/mL of tazobactam in the continuous infusion simulation.

**Figure 1:**
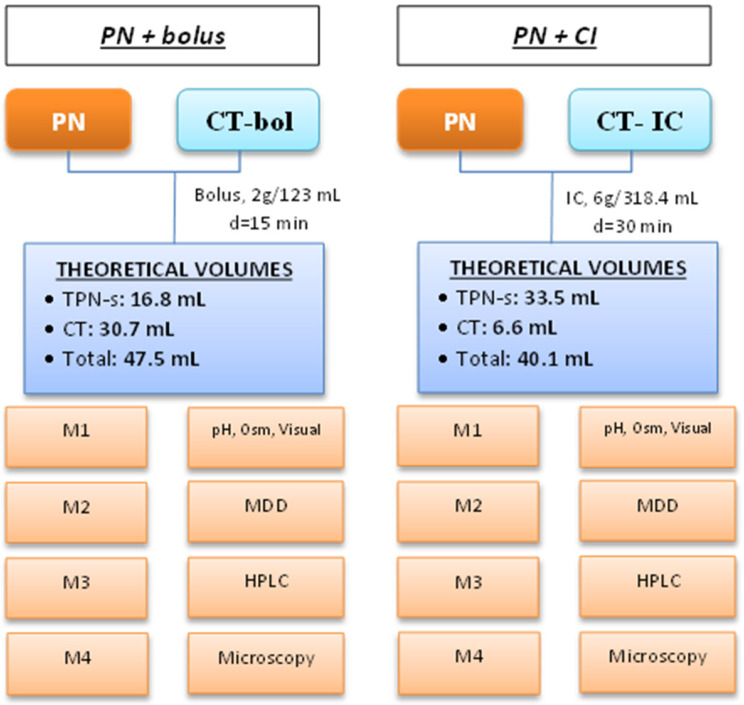
Schema of the study design. Each simulation was triplicated. Abbreviations: Bol: bolus; CI: continuous infusion; CT: ceftolozane-tazobactam; d: infusion duration; M: sample; MDD: MDD sample; pH, Osm, Visual: pH, osmolality and visual inspection sample; HPLC: HPLC–HRMS sample; Microscopy: microscopic inspection sample; PN: 3-in-1 parenteral nutrition. Doses are expressed as doses of ceftolozane.

Figure 1 shows the study design. Forty-eight samples with a final volume of 5 mL were collected in polystyrene test tubes (REF 55475, Sarstedt AG & Co, Nümbrecht, Germany). Twelve samples were used for the high-performance liquid chromatography–high-resolution mass spectrometry (HPLC–HRMS) test, twelve for dynamic light scattering (DLS), twelve for pH measurement, osmolality, and visual inspection, and twelve for microscopic inspection. One blank from each PN was collected in BD syringes (REF 2211085, Becton Dickinson and Company, Madrid, Spain).

Samples were stored at room temperature (18–25 °C) and protected from light, simulating clinical practice. pH measurements and visual and microscopic inspection were performed at zero time and 24 h after sample collection. Osmolality was measured at zero time and 6 h after obtaining the sample. DLS was carried out 6 h after obtaining the samples, and HPLC–HRMS tests were performed at zero time and 24 h after sample collection.

### 4.5. Stability Assessment

To assess Y-site compatibility between CT and PN, we performed the following physical and chemical tests.

**Figure 2:**
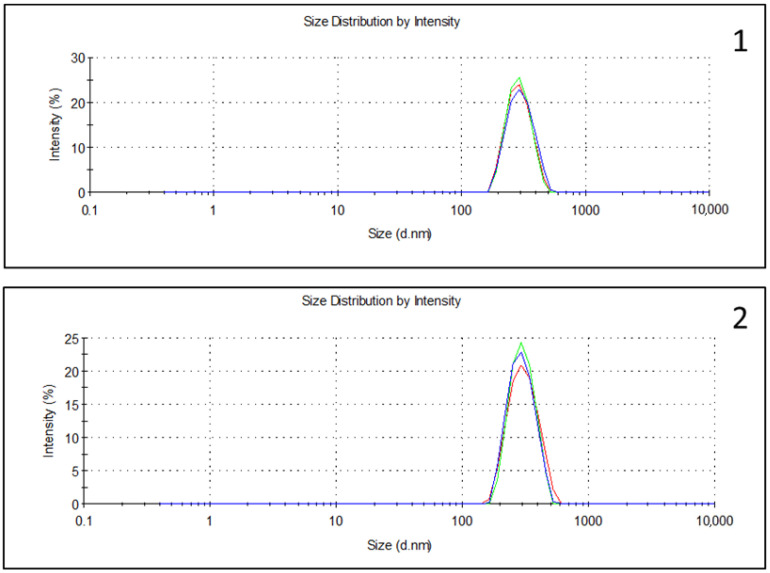
Mean droplet diameter for parenteral nutrition (Green: PN1, Red: PN2, Blue: PN3) with ceftolozane/tazobactam in bolus (**1**) and continuous infusion (**2**).

**Figure 3:**
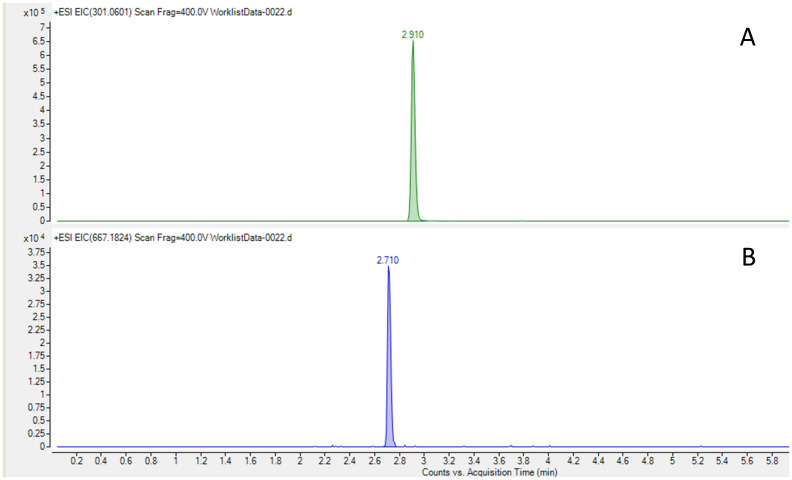
Chromatograms of tazobactam (**A**) and ceftolozane (**B**) obtained through HPLC–HRMS.

- The presence of macroscopic precipitates, changes in color, and phase separation were assessed according to the European Pharmacopeia [35]. Visual inspection was conducted against a black-and-white contrast background by two pharmacists. Visual inspection was also performed with PN emulsions, without the drug.
- Microscopy was assessed using a LEICA DM2500 LED microscope (Leica Microsistemas S.L.U. L’Hospitalet de Llobregat, Spain) by two observers at t = 0 h and t = 24 h. Ten µL of the mixture were assessed at 400× magnification (10× ocular lens and 40× objective lens). Admixtures with CT as a bolus and as continuous infusion concentrations were used as negative control solutions. We also assessed PN with no drug. PN with final unstable calcium and phosphate concentrations was used as a positive control. Each combination of drug and PN was prepared in triplicate.
- pH measurement was made using potentiometry (senIONTM+ PH 1, Hach, Spain) at room temperature.
- Osmolality was measured at room temperature (Osmo1, Advanced Instruments, Tecil, Spain).
- The particle size of the lipid emulsion was measured at 25 °C using dynamic light scattering (Zetasizer NanoZS90, Malvern Instruments Ltd., Malvern, UK) 6 h after the simulation. Samples for DLS were prepared by diluting the solutions into PBS at a final concentration of approximately 30 μg/mL. The results of particle diameter are presented as MDD. (Figure 2 and Supplementary Materials Figure S1)

- HPLC–HRMS was used to quantify CT concentration in the admixtures. Samples were measured in triplicate. An Infinity II LC system coupled to a 6560-ion mobility QTOF mass spectrometer, both from Agilent Technologies (Santa Clara, CA, USA), was used in positive ionization mode from *m*/*z* 100–1700. A Kinetex F5 150 × 2.1 mm, 2.6 µm column from Phenomenex (Torrance, CA, USA) was used with mobile phase A (water and 0.1% formic acid and mobile phase B (Acetonitrile and 0.1% formic acid) at 0.4 mL/min, following the gradient (t(min), %B): (0, 2), (1, 2), (6, 95), (7.5, 95), (7.8, 2), and (10, 2). Samples were diluted 1/10,000 with ultrapure water in three steps and centrifuged at 10,000 rpm at 4 °C for 5 min. The injection volume was 2 µL. Trace chromatograms were extracted with a 3 ppm error at *m*/*z* 667.1824 for ceftolozane and *m*/*z* 301.0601 for tazobactam. (Figure 3) Calibration standards were prepared in a blank matrix diluted 1/10,000, and the weighting of calibration curves was adjusted (1/x or 1/x^2^) to obtain accuracies between 80 and 120%. The matrix-matched calibration curve was prepared in a blank sample diluted 1/10,000, which was spiked with standards at five concentrations between 0.1 and 4.5 mg/L. The calibration curve was prepared daily and was injected in triplicate each day of analysis. Regarding selectivity, the ability to distinguish the analyte from other substances was indicated by an absence of the respective peaks at the same retention time as the corresponding standards in trace chromatograms extracted with a 5 ppm error. Accuracy was calculated at the five spiked levels (based on the ratio between the calculated concentration and the spiked concentration) between 90.5 and 101.2% for tazobactam and between 82.6 and 111.1% for ceftolozane. Intra-day repeatability expressed as relative standard deviation (%) between 0.4 and 3.4% for ceftolozane and between 0.1 and 3.3% for tazobactam was obtained. Inter-day repeatability was between 2.1 and 12.8% for ceftolozane and between 0.8 and 15.9% for tazobactam. The limit of detection LOD, defined as S/N = 3, was 10 ng/mL for ceftolozane and 1 ng/mL for tazobactam.

CT was considered compatible with PN when the following criteria were met:

- No changes in visual inspection, defined as a homogeneous admixture with no changes in color, no phase separation, and lack of macroscopic precipitates or gas formation altering the admixture, as assessed by two independent observers against a black-and-white contrast background at t = 0 h and t = 6 h [35].
- No signs of precipitation, emulsion disruption, or presence of particles >5 µm at microscopic inspection.
- No relevant pH changes at room temperature, defined as a pH between 5.5 and 7.2 in the admixtures at t = 0 and t = 24 h and ΔpH ≤ 0.2 [16,19,24].
- No relevant osmolality changes at room temperature, defined as less than 5% of change between t = 0 h and t = 6 h [19,23].
- No relevant CT concentration changes in the mixture, defined as <20% change at t = 24 h (measured using HPLC–HRMS) [15].
- No relevant particle size changes, defined as MDD ≤ 500 nm in all samples (United States Pharmacopeia), and percentage of particles greater than 5 µm < 0.05% according to the US pharmacopeia method I [38].

Each sample was measured in triplicate, and the results were expressed as the average ± standard deviation.

### 4.6. Data Analysis and Statistics

The CT concentration was calculated using Analyst 1.4.2© Software (AB Sciex, Framingham, MA, USA). MDD, pH, osmolality, and CT results are presented as mean ± SD (Excel© software; Microsoft Office, Redmond, WA, USA, 2017).

## 5. Conclusions

Our findings show the physicochemical compatibility between CT and our PN during Y-site administration, whether as a bolus or continuous infusion. In light of our results, patients with complex drug regimens could benefit from the Y-site administration of CT with PN.

## Figures and Tables

**Table 1:** Results of MDD in simulated admixtures.

Sample	MDD
PN1	276.1 ± 7.7 nm
PN2	286.8 ± 4.7 nm
PN3	284.0 ± 6.5 nm
PN1-bolus1	286.5 ± 8.9 nm
PN2-bolus2	284.7 ± 4.6 nm
PN3-bolus3	282.3 ± 6.3 nm
PN1-IC1	279.2 ± 4.0 nm
PN2-IC2	285.4 ± 2.8 nm
PN3-IC3	294.2 ± 7.3 nm

Notes: PNx refers to the admixtures with no drug, PNx-bolusx refers to the admixture with CT administered as a bolus infusion, and PNx-ICx refers to CT administered in a continuous infusion. MDD, Mean droplet diameter; PN, Parenteral nutrition; CT, ceftolozane-tazobactam. Measurements were made in triplicate.

**Table 2:** Results of osmolality in simulated admixtures.

Sample	Osmolality (mOsm/kg) t = 0 h	Osmolality (mOsm/kg) t = 6 h	Percentage of Initial Osmolality
PN1	1944.3 ± 31.6	1931.7 ± 21.1	−0.7%
PN2	1940 ± 11.1	1922.7 ± 23	−0.9%
PN3	1920.3 ± 17.1	1910.3 ± 12.1	−0.5%
PN1-bolus1	1171 ± 6.6	1149 ± 2.6	−1.9%
PN2-bolus2	1063.3 ± 7.1	1053.3 ± 4.6	−0.9%
PN3-bolus3	1076.3 ± 21	1060.3 ± 8.6	−1.5%
PN1-IC1	1741 ± 23.4	1714 ± 5.3	−1.6%
PN2-IC2	1808 ± 6.6	1744.3 ± 11.2	−3.5%
PN3-IC3	1695.3 ± 16.7	1709 ± 12.1	+0.8%

Notes: PNx refers to the admixtures with no drug, PNx-bolusx refers to the admixture with CT administered as a bolus infusion, and PNx-ICx refers to CT administered in a continuous infusion. Measurements were made in triplicate.

**Table 3:** Changes in ceftolozane concentration according to HPLC analysis at 0 h and 24 h.

Sample	Concentration (mg/mL) t = 0 h	Concentration (mg/mL) t = 24 h	Percentage of the Initial Concentration
PN1-bolus1	8.64 ± 0.32	9.08 ± 0.57	+5%
PN2-bolus2	8.21 ± 0.53	9.58 ± 0.61	+17%
PN3-bolus3	8.45 ± 0.06	9.44 ± 0.44	+12%
PN1-IC1	1.46 ± 0.05	1.32 ± 0.01	−10%
PN2-IC2	1.45 ± 0.04	1.35 ± 0.01	−7%
PN3-IC3	1.81 ± 0.07	1.83 ± 0.04	+1%

Notes: PNx-bolusx refers to the admixture with CT administered as a bolus infusion, and PNx-ICx refers to CT administered in a continuous infusion.

**Table 4:** Changes in the tazobactam concentration according to HPLC analysis at 0 h and 24 h.

Sample	Concentration (mg/mL) t = 0 h	Concentration (mg/mL) t = 24 h	Percentage of the Initial Concentration
PN1-bolus1	4.25 ± 0.10	4.16 ± 0.04	−2%
PN2-bolus2	4.33 ± 0.23	4.37 ± 0.01	+1%
PN3-bolus3	4.32 ± 0.11	4.09 ± 0.28	−5%
PN1-IC1	0.78 ± 0.01	0.78 ± 0.01	0%
PN2-IC2	0.80 ± 0.02	0.77 ± 0.04	−4%
PN3-IC3	1.09 ± 0.05	1.07 ± 0.02	−2%

Notes: PNx-bolusx refers to the admixture with CT administered as a bolus infusion, and PNx-ICx refers to CT administered in a continuous infusion.

**Table 5:** Changes in the ceftolozane–tazobactam ratio according to HPLC analysis results at 0 h and 24 h.

Sample	Concentration Ratio t = 0 h	Concentration Ratio t = 24 h	Percentage of the Initial Concentration Ratio
PN1-bolus1	2.03	2.18	+7%
PN2-bolus2	1.89	2.19	+16%
PN3-bolus3	1.96	2.31	+18%
PN1-IC1	1.87	1.70	−9%
PN2-IC2	1.81	1.75	−3%
PN3-IC3	1.66	1.71	+3%

Notes: PNx-bolusx refers to the admixture with CT administered as a bolus infusion, and PNx-ICx refers to CT administered in a continuous infusion.

**Table 6:** Composition of the Parenteral Nutrition Emulsion.

	PN
Volume (mL)	1615
Total calories (kcal)	1850
Non-protein calories (kcal)	1500
Non-protein calories/gN Ratio	107
Glucose (g)	250
Aminoacids (g)	87.5
Nitrogen (g)	14
Lipids (g)	50
Na^+^ (mEq)	80
K^+^ (mEq)	60
Ca^2+^ (mEq)	9.2
Mg^2+^ (mEq)	10
Phosphate (mEq)	20
Sulphate (mEq)	5
Chlorate (mEq)	60
Acetate (mM)	50
Multivitamin Cernevit^® a^	5 mL
Trace elements Supliven^® b^	10 mL

Notes: PN, parenteral nutrition. ^a^ Multivitamin Cernevit (5 mL): vitamin A (retinol) 3500 IU, vitamin D3 220 IU, vitamin E (α tocopherol) 11.2 IU, vitamin C 125 mg, vitamin B1 (thiamine) 3.51 mg, vitamin B2 (riboflavin) 4.14 mg, vitamin B6 (pyridoxine) 4.53 mg, vitamin B12 6 μg, folic acid 414 μg, pantothenic acid 17.25 mg, biotin 69 μg, vitamin PP (niacin) 46 mg. ^b^ Trace elements Supliven (10 mL): zinc 77 μmol, copper 6 μmol, manganese 1 μmol, selenium 1 μmol, iodine 1 μmol, chromium 0.2 μmol, molybdenum 0.2 μmol, iron 20 μmol, fluorine 50 μmol.

## Data Availability

Data are contained within the paper and the Supplementary Materials.

## Supplementary Materials

The following supporting information can be downloaded at https://www.mdpi.com/article/10.3390/ph17070896/s1, Figure S1: MDD for PN with no drug; Figure S2: MDD for PN and CT bolus infusion; Figure S3: MDD for PN and CT continuous infusion.
