# Supplementary material for: Physicochemical Compatibility of Ceftolozane-Tazobactam with Parenteral Nutrition
Source: Pharmaceuticals (Basel). 2024 Jul 5;17(7):896. doi: 10.3390/ph17070896 (PMC11279994; doi:10.3390/ph17070896)

Supplementary Material

Figure S1. Mean droplet diameter (MDD) for PN with no drug

PN1 (Green: 1st replicate, Red: 2nd replicate, Blue: 3rd replicate)

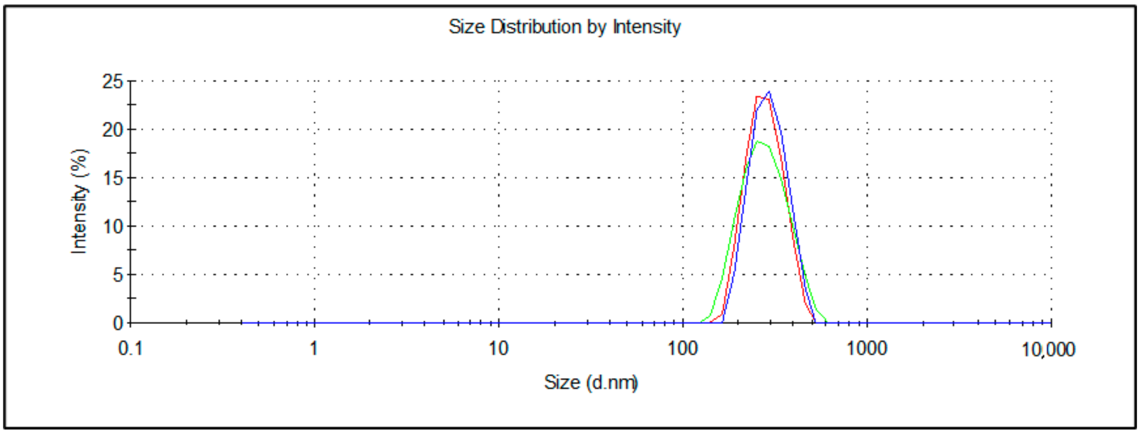

PN2 (Green: 1st replicate, Red: 2nd replicate, Blue: 3rd replicate)

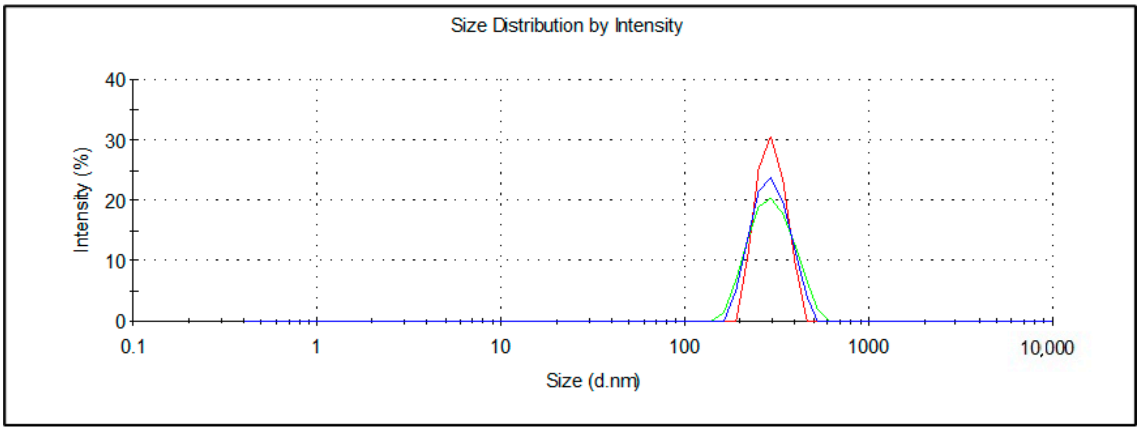

PN3 (Green: 1st replicate, Red: 2nd replicate, Blue: 3rd replicate)

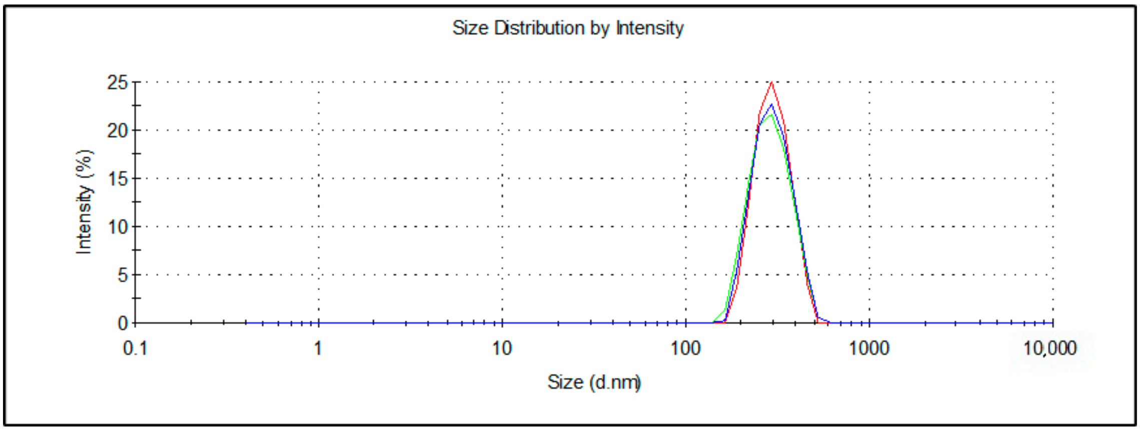

Figure S2. Mean droplet diameter (MDD) for PN and CT bolus infusion

PN1-bolus (Green: 1st replicate, Red: 2nd replicate, Blue: 3rd replicate)

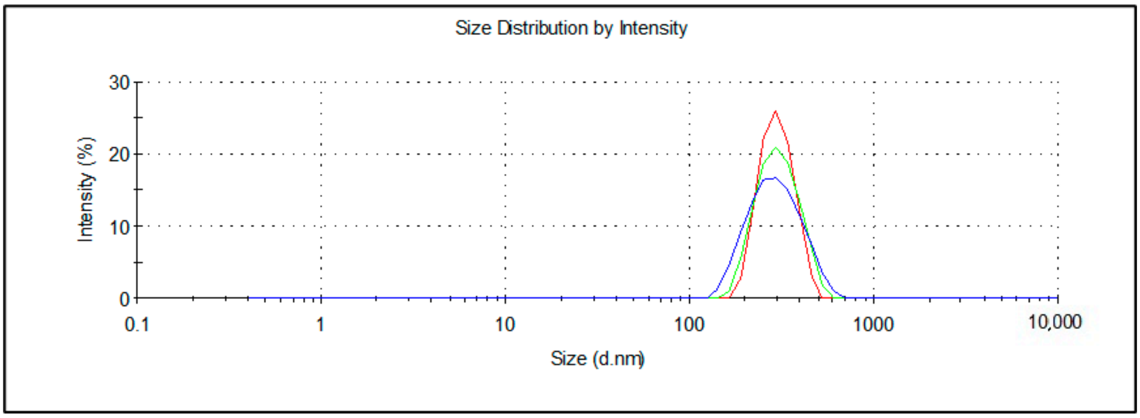

PN2-bolus (Green: 1st replicate, Red: 2nd replicate, Blue: 3rd replicate)

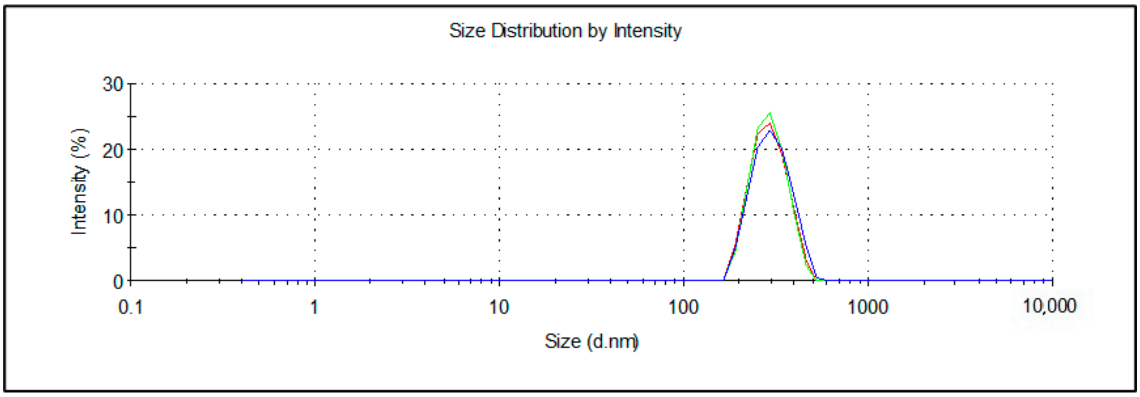

PN3-bolus (Green: 1st replicate, Red: 2nd replicate, Blue: 3rd replicate)

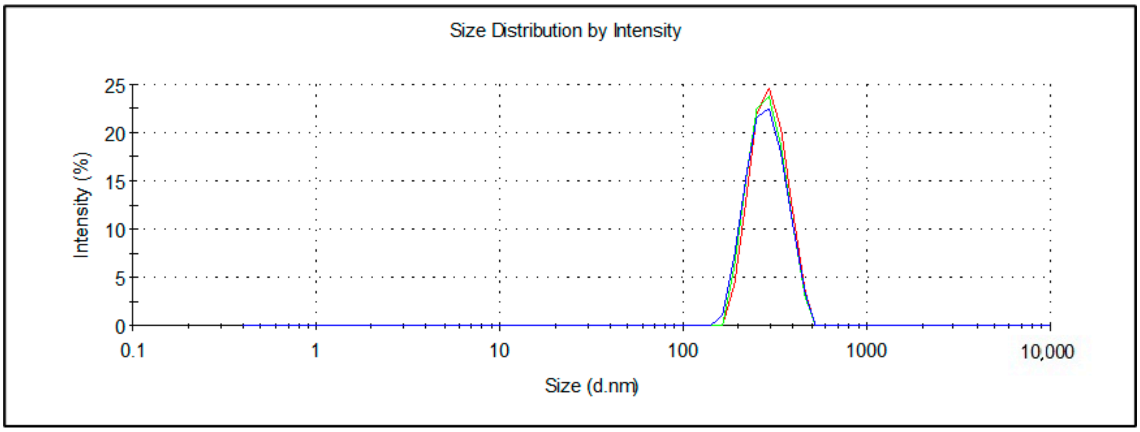

Figure S3. Mean droplet diameter (MDD) for PN and CT continuous infusion

PN1-IC (Green: 1st replicate, Red: 2nd replicate, Blue: 3rd replicate)

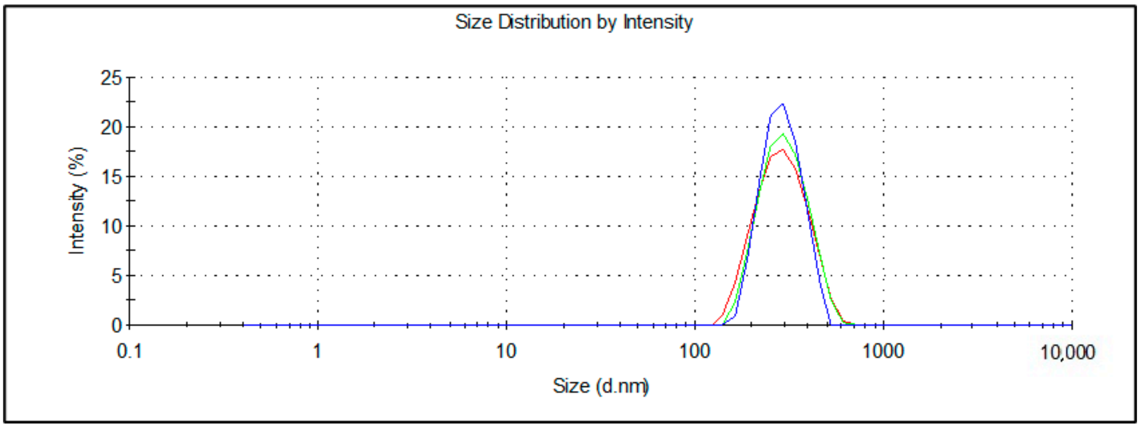

PN2-IC (Green: 1st replicate, Red: 2nd replicate, Blue: 3rd replicate)

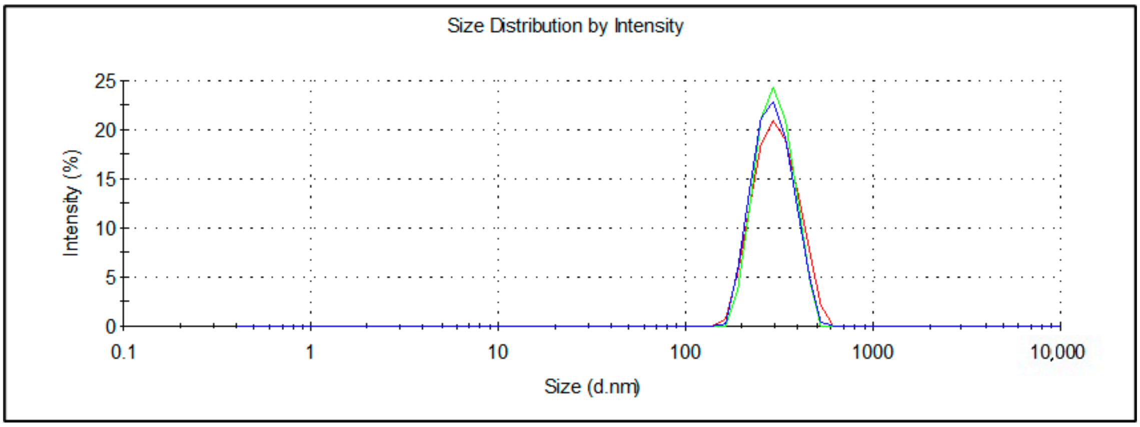

PN3-IC (Green: 1st replicate, Red: 2nd replicate, Blue: 3rd replicate)

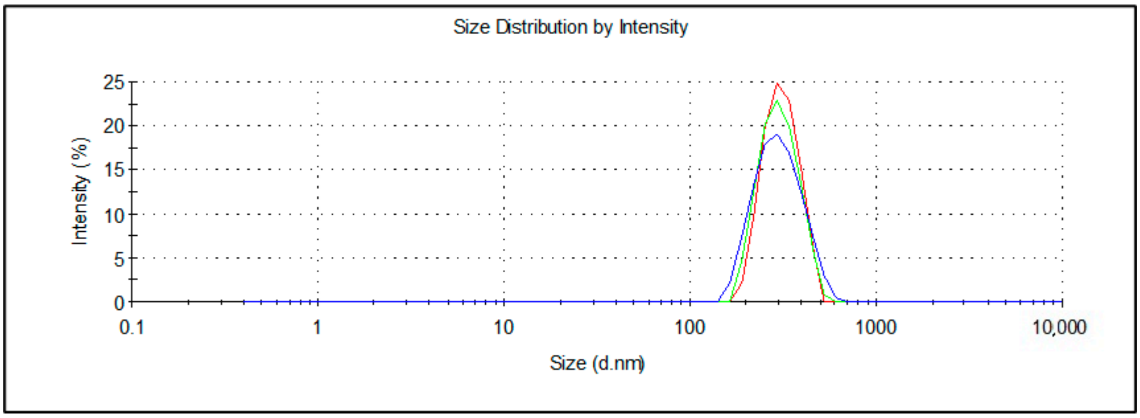

Supplement: Supplementary file 1 [file pharmaceuticals-17-00896-s001.zip › pharmaceuticals-3076395-supplementary.pdf]
